# Spatially fractionated radiotherapy combined with CapeOX and bevacizumab for postoperative pulmonary metastasis from urachal carcinoma: a case report

**DOI:** 10.3389/fonc.2025.1688215

**Published:** 2025-11-17

**Authors:** Jian Fang, Aixin Wang, Quan Yao, Xinyi Liu, Weiyi Zhou, Jinyi Lang

**Affiliations:** 1 Department of Oncology, The Affiliated Hospital of Southwest Medical University, Luzhou, Sichuan, China; 2 Department of Radiation Oncology, Radiation Oncology Key Laboratory of Sichuan Province, Sichuan Clinical Research Center for Cancer, Sichuan Cancer Hospital & Institute, Sichuan Cancer Center, University of Electronic Science and Technology of China, Chengdu, Sichuan, China

**Keywords:** spatially fractionated radiotherapy, lattice radiotherapy, urachal carcinoma, signet ring cell carcinoma, pulmonary metastasis

## Abstract

Urachal carcinoma is a rare and aggressive malignancy with limited treatment options, particularly in the metastatic setting. Standardized therapeutic protocols are lacking. This case report describes a 62-year-old female patient who developed pulmonary and mediastinal lymph node metastases more than four years after surgical resection of urachal carcinoma. For this case, a combined regimen of Spatially Fractionated Radiotherapy (SFRT), CapeOX (capecitabine plus oxaliplatin) chemotherapy, and bevacizumab targeted therapy was developed. The treatment course was complicated by grade 4 hematologic toxicity and radiation-induced esophagitis, both of which were successfully managed. To our knowledge, this represents the first documented application of SFRT in urachal carcinoma. Although significant tumor shrinkage was not observed, the patient achieved symptomatic relief, metabolic remission, and stable disease during follow-up.

## 1 Introduction

Urachal carcinoma is a rare and highly aggressive malignancy arising from embryonic urachal remnants. Its estimated annual incidence is approximately one per million, accounting for only 0.35%–0.7% of all bladder cancers (1). The predominant histology is adenocarcinoma (>90%) (2). Among these, signet ring cell carcinoma is a rare subtype, accounting for only 7% (3). Owing to its unique origin and biological behavior, diagnosis often occurs at advanced stages, leading to high rates of post-treatment local recurrence or distant metastasis. The lung is a common site of metastasis (4). Currently, there is no standardized treatment protocol for urachal carcinoma with pulmonary metastases. Management typically involves surgical resection combined with systemic chemotherapy, yet the tumor is generally considered radioresistant (5). Spatially fractionated radiotherapy (SFRT) is an advanced radiotherapeutic technique that delivers ablative doses to large tumors while sparing surrounding normal tissues (6). Initially described with GRID collimation in 1909 and refined in the 1950s, this approach has since evolved into three-dimensional Lattice radiotherapy. Although SFRT has shown promise in other malignancies (7–10), its application in urachal carcinoma has not been reported. Here, we present the first documented case of postoperative pulmonary and mediastinal lymph node metastases from urachal carcinoma treated with SFRT in combination with CapeOX chemotherapy and bevacizumab.

## 2 Case presentation

**Figure 1:**
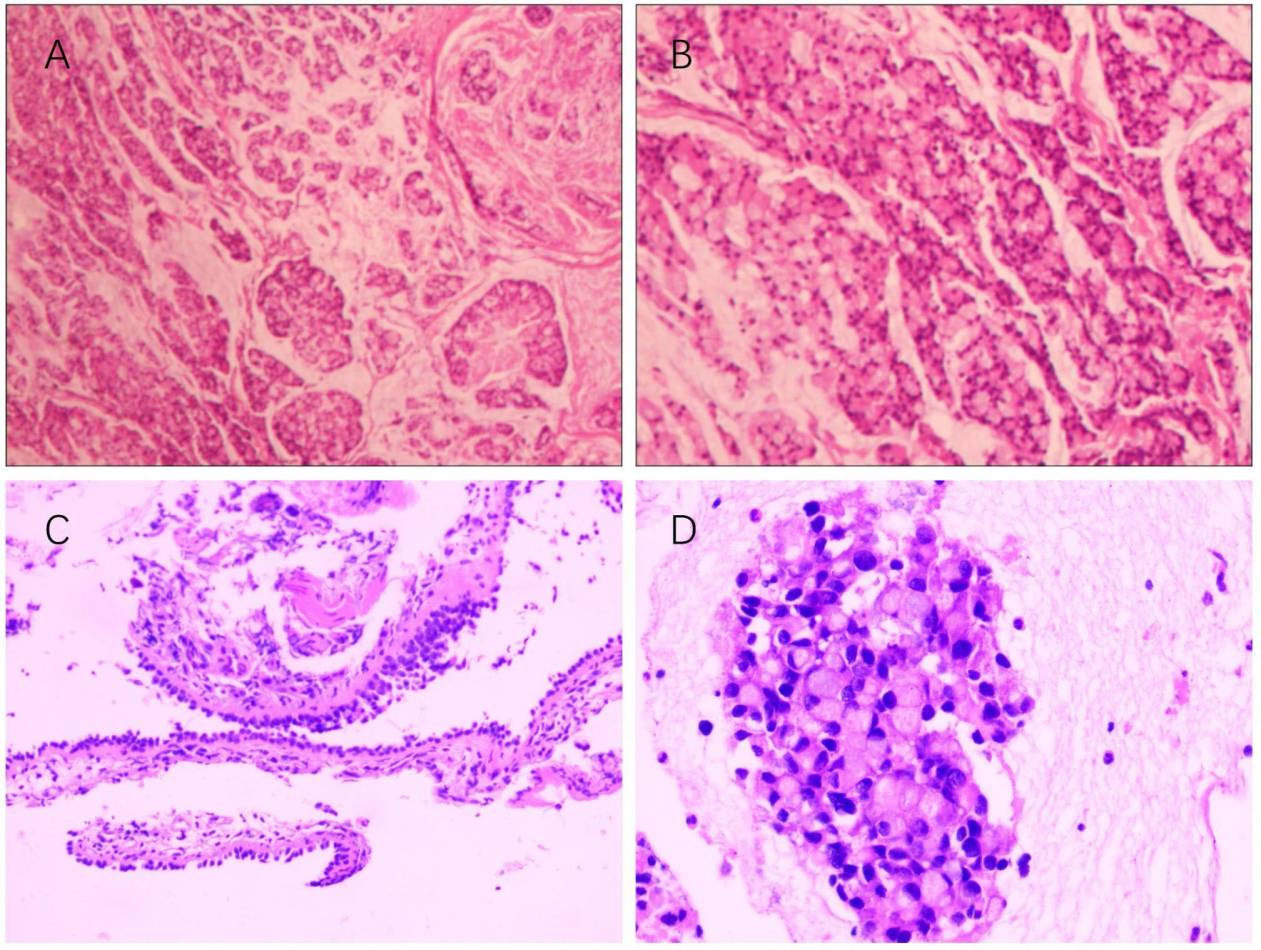
**(A, B)** Histopathological features of the primary tumor (surgical resection specimen, 2020). Hematoxylin and eosin staining; magnification ×10 and ×20. **(C, D)** Histopathological features of the metastatic site (Station 11R lymph node, 2025). Hematoxylin and eosin staining; magnification ×100 and ×400.

**Figure 2:**
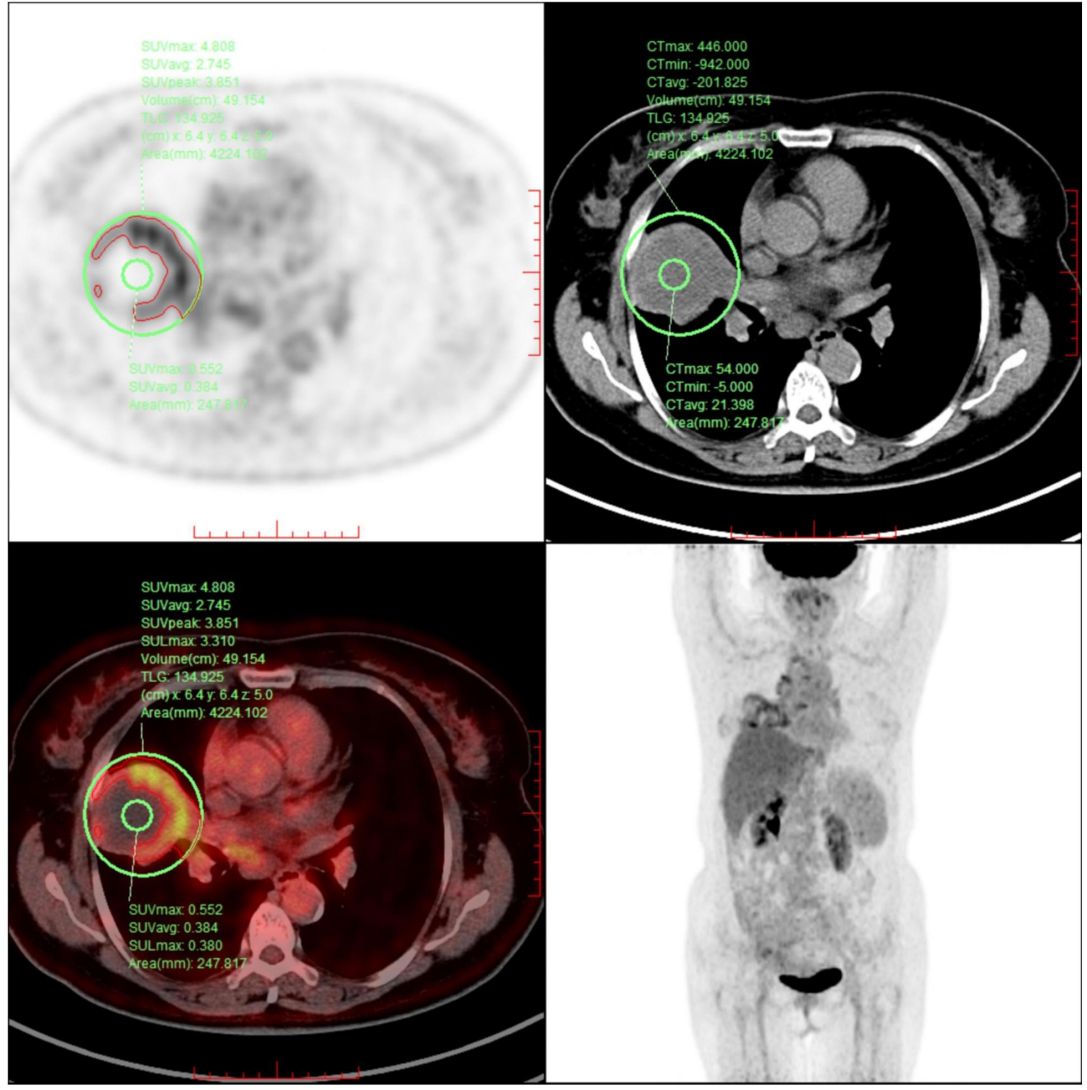
The image reveals a ring-shaped pattern of increased FDG uptake, with higher standardized uptake values (SUV) observed at the periphery of the lesion compared to its center.

This case report presents a 62-year-old female patient with urachal carcinoma who underwent partial cystectomy and en bloc resection of the urachal tumor at West China Hospital of Sichuan University in May 2020. Histopathological examination revealed moderately to poorly differentiated adenocarcinoma (Sheldon stage IIIA). Pathological specimens reviewed at our institution confirmed mucinous adenocarcinoma with signet ring cell components (**Figure 1**). Immunohistochemistry demonstrated CK20(+), PAX-8(–), CK7(–), P63(–), GATA-3(–), CDX-2(+), β-catenin (+), p53 (wild-type), and Ki-67 (~5% positive). Postoperatively, the patient received one cycle of capecitabine plus cisplatin chemotherapy. Due to severe adverse effects, the regimen was switched to four cycles of oral capecitabine monotherapy. Regular follow-up over two years revealed no evidence of recurrence. On December 14, 2024, the patient presented with unexplained chest tightness accompanied by cough and chest pain. On December 24, 2024, she was admitted to our hospital for further diagnostic evaluation and treatment. Imaging revealed a cystic-solid mass in the right middle lobe, measuring 6.8 × 6.3 cm, alongside multiple enlarged mediastinal lymph nodes. Fluorine-18 fluorodeoxyglucose positron emission tomography/computed tomography (^18^F-FDG PET-CT) demonstrated a maximum standardized uptake value (SUVmax) of 4.8 in the solid component of the right lung mass (**Figure 2**). Upper gastrointestinal endoscopy and colonoscopy revealed chronic atrophic gastritis and ascending colon polyps. Abdominal contrast-enhanced MRI showed no definite abnormalities at the previous surgical site. Ultrasound-guided bronchoscopy with fine-needle aspiration biopsy was performed to obtain a definitive diagnosis. Histopathological examination of the biopsy specimen confirmed metastatic urachal adenocarcinoma with signet ring cell morphology (**Figure 1**). The immunophenotype of both primary and metastatic lesions (CK20+/CDX-2+/CK7–) supported a diagnosis of metastatic urachal carcinoma. Absence of GATA-3, TTF-1, and Napsin A excluded the possibility of primary lung cancer (5).

**Figure 3:**
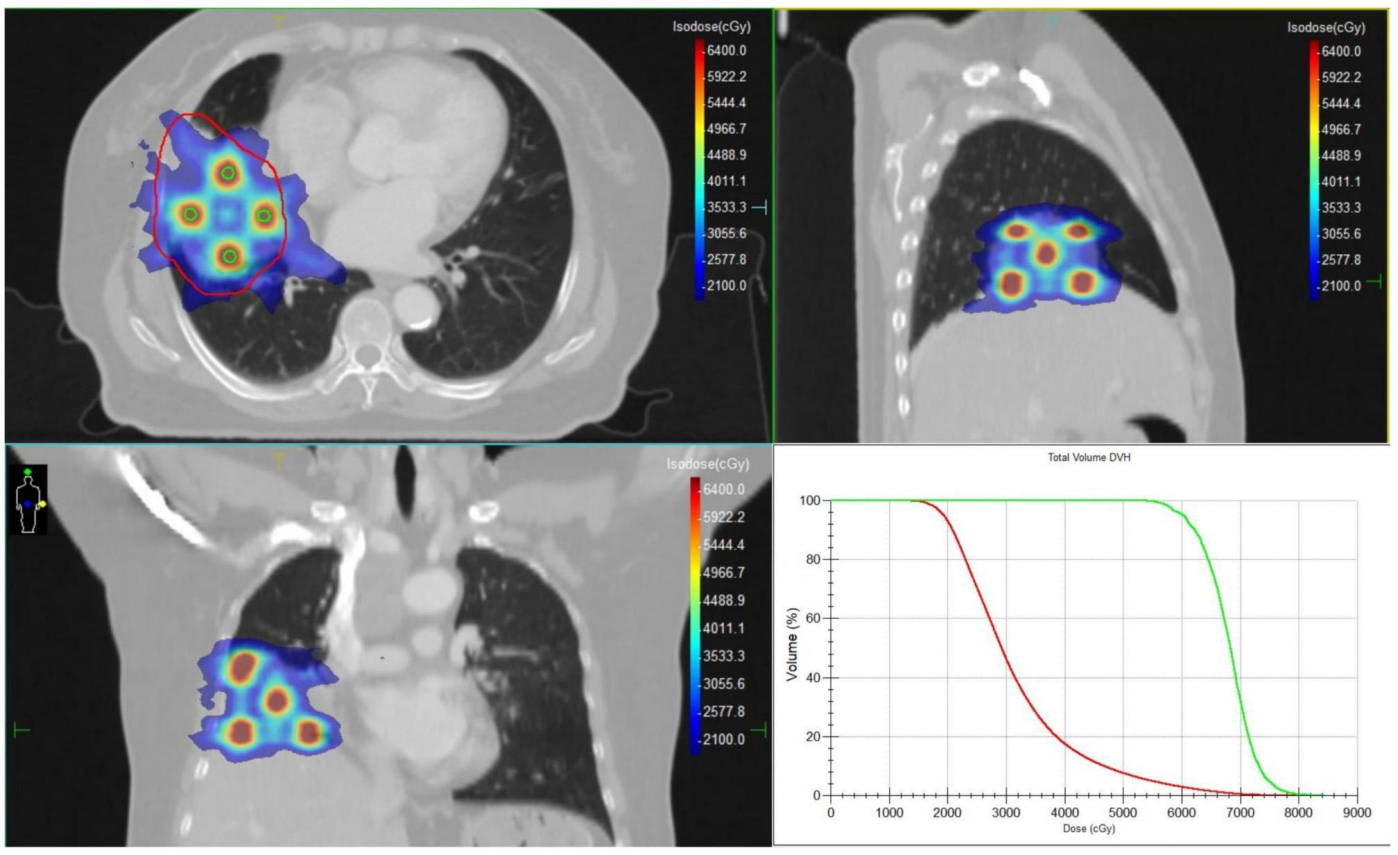
The target volumes and dose distribution of the SFRT, and the dose-volume histograms (DVHs) for GTV and GTV-peak. The red contour represents the GTV, and the green contour represents the GTV-peak.

Among all tumor markers assessed, CA72–4 was markedly elevated at 241.93 U/mL (reference range: 0–7.0 U/mL). Following multidisciplinary consultation, surgical intervention was deemed infeasible. Given the histologic and biological similarities between colorectal adenocarcinoma, chemotherapy regimens are mostly based on colorectal adenocarcinoma. Due to the patient’s intolerance to prior cisplatin plus capecitabine chemotherapy, CapeOX plus bevacizumab was chosen as systemic therapy, which has been documented and shown good efficacy (11). Between February 5 and March 23, 2025, the patient underwent a comprehensive radiotherapy regimen targeting pulmonary metastases and involved mediastinal lymph nodes. All treatment plans were generated using the Monaco treatment planning system, with meticulous delineation of the gross tumor volume (GTV) and organs at risk (OAR). In total, eight treatment plans were developed over the course of therapy. Each plan underwent independent review by both the attending radiation oncologist and a medical physicist to ensure dosimetric accuracy and clinical safety. For the right lung lesion, Lattice SFRT was delivered using multileaf collimator (MLC)-based volumetric modulated arc therapy (VMAT). Given the proximity of the lesion to the heart, a dedicated subvolume (GTV-violet) was contoured to safeguard cardiac structures, while the remaining tumor volume was defined as GTV-red. Within GTV-red, spherical high-dose vertices (GTV-peak) were arranged according to a hexagonal close-packed model. Each vertex measured 0.4 cm in diameter, with a center-to-center spacing of 2.0 cm and an interplanar spacing of 2.0 cm. All vertices were positioned at least 1 cm from the tumor margin and adjacent critical organs. **Figure 3** illustrates the spatial distribution of GTV-peaks and the corresponding dose distribution. A total of four SFRT sessions were delivered. In sessions 1 and 2, the GTV-peak dose was 10.5 Gy and the low-dose zone received 3.5 Gy, whereas in sessions 3 and 4, the GTV-peak dose was 12 Gy and the low-dose zone received 4 Gy. The dose ratio between the high-dose and low-dose regions was consistently 3:1 for all sessions. Following SFRT to the right lung lesion, adjuvant VMAT was delivered. For the mediastinal metastatic lymph nodes, Partial Ablative Body Radiotherapy (PABR), another form of SFRT, was employed. In PABR, an escalated ablative dose is administered to the central tumor region, whereas a reduced dose is prescribed to the peripheral margins. The prescribed central high-dose and peripheral low-dose levels were as follows:Station 3 lymph node: 10 Gy (center), 3 Gy (periphery); Station 4 lymph node: 8 Gy (center), 4 Gy (periphery); Station 7 lymph node: 8 Gy (center), 3 Gy (periphery). VMAT was subsequently administered following PABR. All VMAT regimens adopted an accelerated fractionation schedule, consisting of two fractions per day with an inter-fraction interval exceeding 6 hours. Dose constraints for organs at risk adhered to the American Association of Physicists in Medicine (AAPM) Task Group 101 guidelines for 5-fraction SBRT. Treatments were delivered using an Elekta Infinity 6 MV linear accelerator, with cone-beam CT (CBCT) performed prior to each session to verify tumor positioning. Details of the radiotherapy workflow and fractionation schedules are provided in **Supplementary Table S1**.

**Figure 4:**
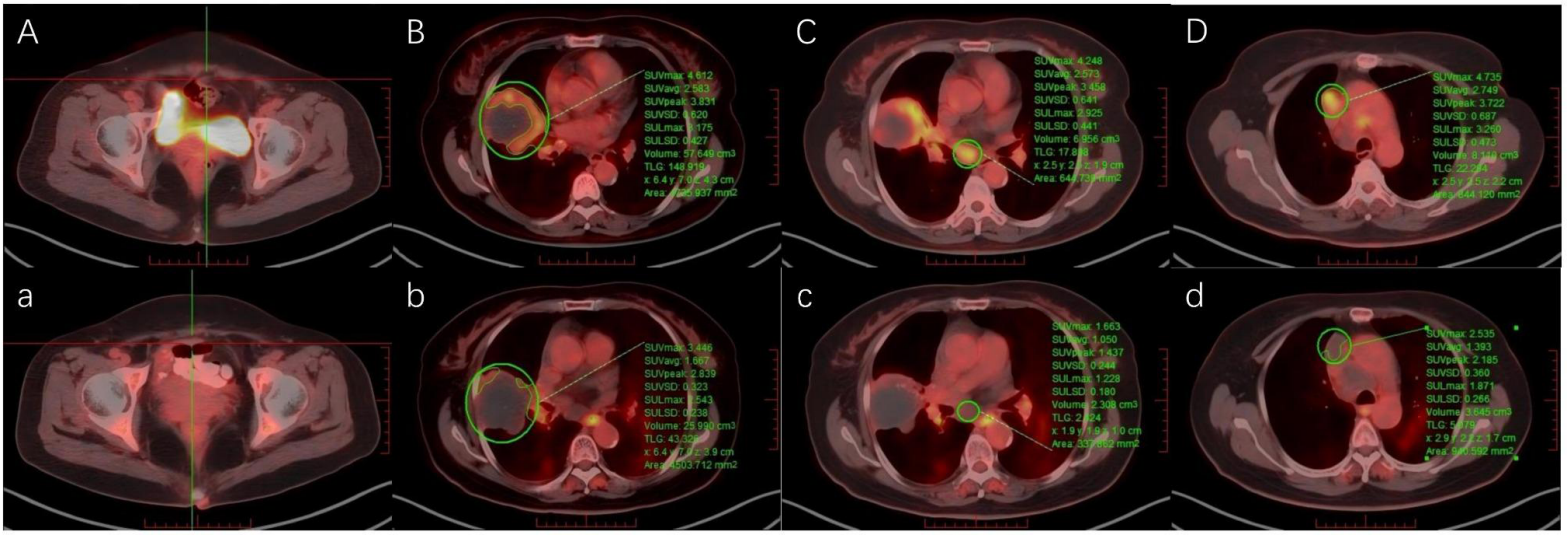
Pre-treatment and post-treatment ^18^F-FDG PET-CT images of **(A, a)** the surgical site, **(B, b)** the right lung lesion, **(C, c)** mediastinal lymph nodes in station 7, and **(D, d)** mediastinal lymph nodes in station 3.

On April 5, 2025, after three cycles of chemotherapy plus targeted therapy, the patient developed grade 4 bone marrow suppression (Common Terminology Criteria for Adverse Events, version 5.0; CTCAE v5.0), with a platelet nadir of 24 × 10^9^/L. Management included recombinant human thrombopoietin (rhTPO) and platelet transfusions, resulting in hematologic recovery. Subsequently, the patient chose to discontinue anticancer therapy. One month after completion of treatment, CA72-4 levels had decreased to 48.02 U/mL. A follow-up ^18^F FDG PET-CT scan on May 7, 2025, indicated stable disease (SD) according to Response Evaluation Criteria in Solid Tumours version 1.1 (RECIST 1.1). However, a reduction in the SUVmax of the tumor lesions was noted compared with baseline. Increased FDG uptake was noted in a segment of the esophagus (**Figure 4**). Upper gastrointestinal endoscopy confirmed an ulcer 22 cm from the incisors (CTCAE grade 3, v5.0). Since admission, the patient’s cough and chest tightness caused by the tumor had improved significantly. October 25,2025, the patient remains under follow-up.

## 3 Discussion

Given the extensive tumor burden in the right lung and mediastinal lymph nodes, surgical resection was not feasible. Pathological examination further revealed a signet-ring cell carcinoma component. This subtype is typically more aggressive, has higher proliferative potential, and shows reduced sensitivity to both radiotherapy and chemotherapy, resulting in a poorer prognosis. Moreover, the patient had previously demonstrated intolerance to cisplatin plus capecitabine chemotherapy, further limited systemic treatment options. The role of radiotherapy remains uncertain, as urachal carcinoma is generally considered radioresistant. A population-based study using Surveillance, Epidemiology, and End Results (SEER) data indicated that only 10% of patients received radiotherapy (12), primarily as postoperative adjuvant treatment (13). In recent years, SFRT has shown promising efficacy in bulky tumors; therefore, we designed an aggressive palliative radiotherapy regimen to maximize local tumor control.

For the right lung lesion, SFRT was delivered in four sessions, achieving a biologically effective dose (BED, α/β=10) of 168.25 Gy in the high-dose tumor region—significantly exceeding conventional palliative doses and highlighting its dosimetric advantage. The VMAT plan employed an accelerated fractionation schedule. Firstly,for large, rapidly proliferating malignancies, conventional protracted courses of radiotherapy may compromise local control due to accelerated repopulation during treatment (14). The BID regimen was adopted to markedly shorten overall treatment time, consistent with Fowler’s linear-quadratic model analysis, which emphasizes time as a critical determinant of radiotherapy efficacy (15). While pursuing treatment intensification, equal attention was given to normal tissue safety. During BID delivery, an interval of at least 6 hours between fractions was strictly maintained. This approach was informed by established accelerated hyperfractionation protocols such as CHART (16), whose radiobiological rationale lies in allowing normal tissues (e.g., esophagus, lung) sufficient time for sublethal damage repair. Although radiobiological data specific to urachal carcinoma are lacking, the aggressive histology in this case (signet-ring cell carcinoma) implies a high proliferative potential. Extrapolating from paradigms established in other rapidly proliferating tumors, BID fractionation was adopted as a rational intensification strategy. Following completion of radiotherapy, the patient experienced radiation-induced esophagitis, which was subsequently confirmed by clinical evaluation (CTCAE Grade 3, v5.0). Cumulative DVH analysis integrating all radiotherapy plans revealed an esophageal Dmax of 82.88 Gy and Dmean of 26.75 Gy (**Supplementary Figure S1**), exceeding conventional tolerance thresholds. This adverse event was highly consistent with the cumulative esophageal dose from accelerated mediastinal irradiation, an anticipated risk of such an aggressive regimen. Regarding tumor response, PET-CT demonstrated reduced metabolic activity, although lesion volume remained stable. This may be explained by the central region of the lesion being predominantly mucinous, secreted by the tumor, as well as the inherent radioresistance of urachal carcinoma. Nevertheless, achieving metabolic remission and local control in such an inoperable advanced disease remains of significant clinical value.

Given the limited efficacy of both radiotherapy and chemotherapy, novel treatments for urachal carcinoma are urgently needed. Case reports have documented responses to targeted therapies in urachal carcinoma, including *EGFR* inhibitors (gefitinib, cetuximab), *VEGFR* inhibitors (sunitinib), and *PARP*/*MEK* inhibitors (17–21). Immune checkpoint inhibitors (ICIs) have advanced rapidly in oncology; potential biomarkers for urachal carcinoma include mismatch repair (MMR) deficiency and tumor cell PD-L1 positivity. Kardos et al. reported a patient with MSH6-mutated urachal carcinoma who benefited from the anti-PD-L1 antibody atezolizumab (22). However, our patient exhibited PD-L1-negative expression (TPS <1%), potentially limiting the efficacy of ICIs and precluding the selection of immunotherapy. Subsequent testing revealed *KRAS* and *NRAS* wild-type status and no *BRAF* mutation. Immunohistochemistry also revealed high Trop-2 expression as a potential therapeutic target. Antibody–drug conjugates targeting Trop-2 have shown efficacy in breast and lung cancers (23, 24). If the patient seeks further treatment, cetuximab combined with chemotherapy or Trop-2-targeted antibody–drug conjugate therapy may be considered.

## 4 Conclusion

This case represents the first documented use of SFRT combined with CapeOX chemotherapy and bevacizumab in metastatic urachal carcinoma. Although significant tumor volume reduction was not achieved, the treatment resulted in metabolic remission and symptomatic relief in a patient with otherwise limited therapeutic options. Furthermore, this case provides additional evidence for the poor radiosensitivity of urachal carcinoma. Immunohistochemical analysis revealed high Trop-2 expression (3+) in the tumor tissue. Future studies should actively explore novel targeted therapeutic strategies, particularly Trop-2-targeted antibody-drug conjugates, to improve survival in this poor-prognosis malignancy.

## Data Availability

The original contributions presented in the study are included in the article/**Supplementary Material**. Further inquiries can be directed to the corresponding author/s.
