# Supplementary material for: Spatially fractionated radiotherapy combined with CapeOX and bevacizumab for postoperative pulmonary metastasis from urachal carcinoma: a case report
Source: Front Oncol. 2025 Nov 17;15:1688215. doi: 10.3389/fonc.2025.1688215 (PMC12665564; doi:10.3389/fonc.2025.1688215)
Supplement: Supplementary file 1 [file DataSheet1.docx]

Supplementary Material

# Supplementary Tables

**Supplementary Table 1: Summary of Radiotherapy Targets, Treatment Details**

| **Radiotherapy Target** | **Radiotherapy Site** | **Treatment Details** |
| --- | --- | --- |
| 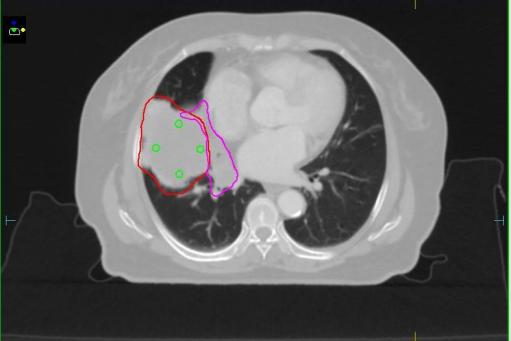 | **GTV-peak** (green)  Right lung lesion away from heart **(GTV-red)**  Right lung lesion adjacent to heart **(GTV-violet)** | **February 5, 2025, February 7, 2025：SFRT**  GTV-peak: 10.5 Gy/F × 2F；GTV-red: 3.5 Gy/F × 2F；GTV-violet: 2.5 Gy/F × 2F  **February 11, 2025, February 14, 2025：VMAT**  GTV-red: 4.0 Gy/F bid× 2d；GTV-violet: 2.5 Gy/F bid × 2d  **February 23, 2025：SFRT**  GTV-peak: 12.0 Gy/F × 1F; GTV-red: 4.0 Gy/F × 1F; GTV-violet: 3.0 Gy/F × 1F  **February 25, 26, 27, 28, 2025, and March 1, 2025：VMAT**  GTV-red: 2.5 Gy/F bid × 5d; GTV-violet: 1.8 Gy/F bid × 5d  **March 7, 2025：SFRT**  GTV-peak: 12.0 Gy/F × 1F; GTV-red: 4.0 Gy/F × 1F; GTV-violet: 3.0 Gy/F × 1F  **March 10, 11, and 13, 2025：VMAT**  GTV-red: 2.5 Gy/F bid × 3d; GTV-violet: 1.8 Gy/F bid × 3d |
| 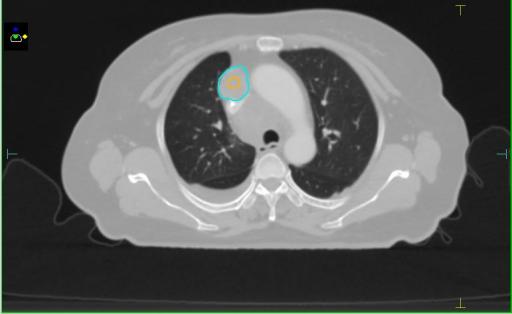 | **Mediastinal lymph node station 3** | **March 3, 6, and 9, 2025：PABR**  3.0 Gy/F bid× 3d  Center: 10 Gy/F bid× 3d |
| 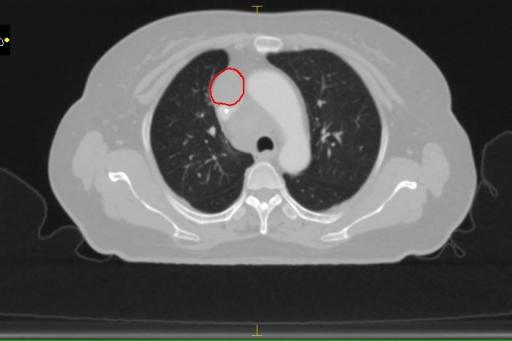 | **Mediastinal lymph node station 3** | **March 21, 2025, March 23, 2025:** **VMAT**  3.0 Gy/F bid × 2d |
| 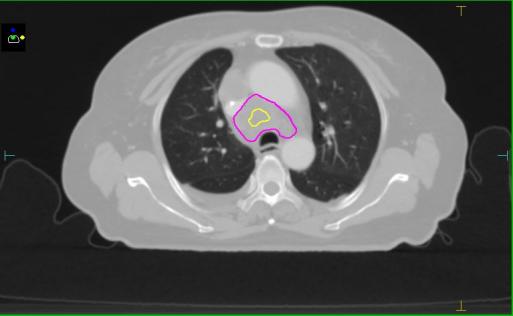 | **Mediastinal lymph node station 4** | **March 3, 6, and 9, 2025：PABR**  4.0 Gy/F bid × 3d  Center: 8 Gy/F bid × 3d |
| 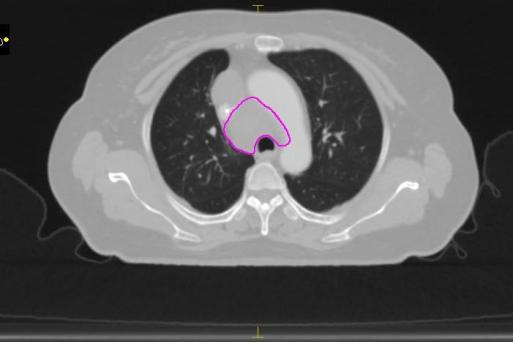 | **Mediastinal lymph node station 4** | **March 21, 2025, March 23, 2025:** **VMAT**  3.0 Gy/F bid × 2d |
| 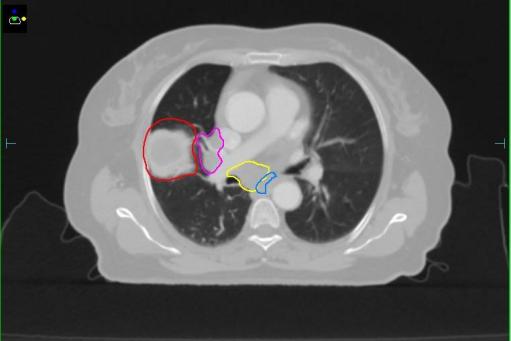 | Mediastinal lymph node station7 away from esophagus (**GTV-yellow**)  Mediastinal lymph node station 7 adjacent to esophagus (**GTV-blue**) | **February 5, 2025, February 7, 2025：SFRT**  GTV-yellow: 2.5 Gy/F × 2F; GTV-blue: 1.5 Gy/F × 2F  **February 11, 2025, February 14, 2025：VMAT**  GTV-yellow: 2.5 Gy/F bid × 2d; GTV-blue: 1.5 Gy/F bid × 2d  **March 21, 2025, March 23, 2025: VMAT**  GTV-yellow: 2.5 Gy/F bid × 2d; GTV-blue: 1.8 Gy/F bid × 2d |
| 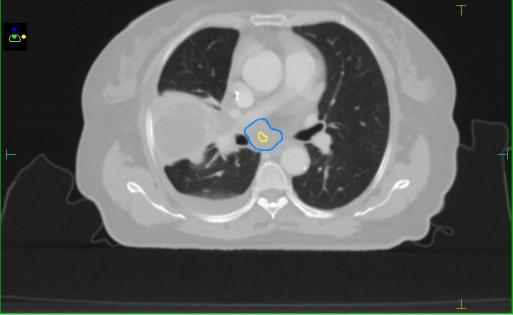 | Mediastinal lymph node station 7 | **March 3, 6, and 9, 2025：PABR**  3.0 Gy/F bid × 3d  Center: 8 Gy/F bid × 3d |

Abbreviation: SFRT: spatially fractionated radiotherapy; VMAT: Volumetric Modulated Arc Therapy; GTV: Gross Tumor Volume. PABR: Partial Ablative Body Radiotherapy

# 2. Supplementary Figure 1

Combined dose–volume histograms (DVHs) for organs at risk (OARs) from all radiotherapy plans


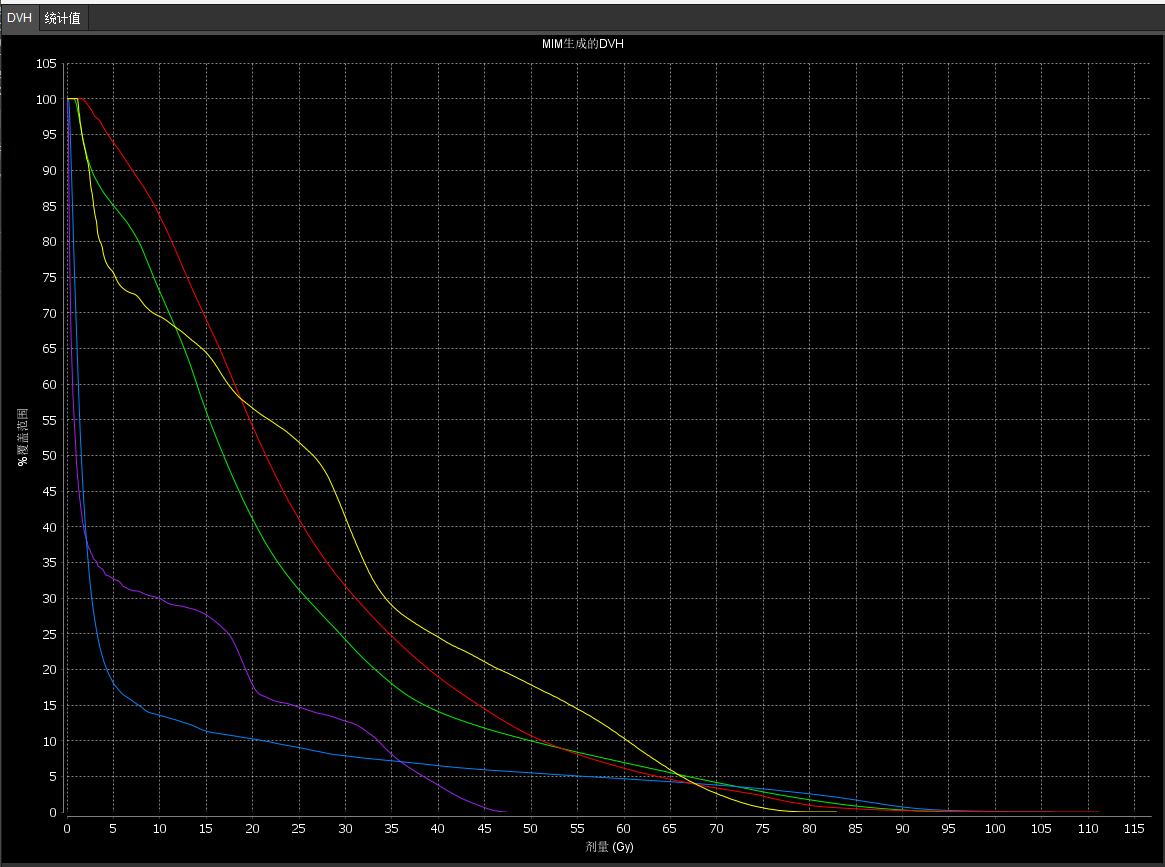


The figure shows the cumulative dose distribution delivered to major organs at risk (OARs) throughout the entire course of radiotherapy. The esophagus is shown in yellow, the heart in red, the lungs in green, the spinal cord in violet, and the liver in blue.
